# Carnitine Palmitoyltransferase 1b Deficient Mice Develop Severe Insulin Resistance After Prolonged High Fat Diet Feeding

**DOI:** 10.4172/2155-6156.1000401

**Published:** 2014-07-04

**Authors:** Teayoun Kim, John F Moore, Jon D Sharer, Kevin Yang, Philip A Wood, Qinglin Yang

**Affiliations:** 1 Department of Nutrition Sciences, University of Alabama at Birmingham, Alabama, USA; 2 Department of Genetics, University of Alabama at Birmingham, Alabama, USA; 3 Sanford-Burnham Medical Research Institute at Lake Nona, Orlando, Florida, USA

**Keywords:** CPT1b, Insulin sensitivity, Skeletal muscle

## Abstract

**Background:**

Carnitine palmitoyltransferase 1 (CPT1) is the rate-limiting enzyme governing the entry of long-chain acyl-CoAs into mitochondria. Treatments with CPT1 inhibitors protect against insulin resistance in short-term preclinical animal studies. We recently reported that mice with muscle isoform CPT1b deficiency demonstrated improved insulin sensitivity when fed a High Fat-Diet (HFD) for up to 5 months. In this follow up study, we further investigated whether the insulin sensitizing effects of partial CPT1b deficiency could be maintained under a prolonged HFD feeding condition.

**Methods:**

We investigated the effects of CPT1b deficiency on HFD-induced insulin resistance using heterozygous CPT1b deficient (*Cpt1b*^+/−^) mice compared with Wild Type (WT) mice fed a HFD for a prolonged period of time (7 months). We assessed insulin sensitivity using hyperinsulinemic-euglycemic clamps. We also examined body composition, skeletal muscle lipid profile, and changes in the insulin signaling pathways of skeletal muscle, liver, and adipose tissue.

**Results:**

We found that *Cpt1b*^+/−^ mice became severely insulin resistant after 7 months of HFD feeding. *Cpt1b*^+/−^ mice exhibited a substantially reduced glucose infusion rate and skeletal muscle glucose uptake. While *Cpt1b*^+/−^ mice maintained a slower weight gain with less fat mass than WT mice, accumulation of lipid intermediates became evident in the muscle of *Cpt1b*^+/−^ but not WT mice after 7 months of HFD feeding. Insulin signaling was impaired in the *Cpt1b*^+/−^ as compared to the WT muscles.

**Conclusion:**

Partial CPT1b deficiency, mimicking CPT1b inhibition, may lead to impaired insulin signaling and insulin sensitivity under a prolonged HFD feeding condition. Therefore, further studies on the potential detrimental effects of prolonged therapy with CPT1 inhibition are necessary in the development of this potential therapeutic strategy.

## Introduction

Diabetes affects 25.8 million people in the United States (CDC 2011 Diabetes Fact Sheet) and 347 million people worldwide (WHO 2013 Diabetes Fact Sheet). Diet-induced obesity and insulin resistance are leading causes of type 2 diabetes. Since imbalance between energy intake and expenditure is a major problem in insulin resistant and type 2 diabetes patients, changes in lifestyle should provide the ultimate solution for these metabolic syndromes [1]. On the other hand, a combination including drug therapy may be essential for insulin resistant, as well as diabetic patients, in which exercise and lifestyle changes are not feasible [1,2].

Carnitine Palmitoyltransferase-1 (CPT1) has recently emerged as an attractive therapeutic target against insulin resistance, based on a theory derived from the Randle Cycle that inhibition of CPT1 activity may promote the redirection of mitochondrial substrate metabolism from fatty acids to glucose, thus ameliorating insulin resistance [3]. Numerous investigations have shown various results on the insulin sensitizing effects of CPT1 inhibitors, such as etomoxir, the dinitrophenol derivative of etomoxir (DNP-etomoxir), oxfenicine (S-2-(4-hydroxylphenyl) glycine), and phenylalkyl oxirane carboxylates (POCA) (reviewed in [4]). Etomoxir improves insulin sensitivity even with increased lipid accumulation in the muscle of humans subjected to 3 days of HFD-feeding followed by 2 days of etomoxir treatment [5]. Oxfenicine improves insulin sensitivity in mice under HFD-feeding for 3 months without intramyocellular lipid accumulation [6]. On the other hand, opposite effects have also been observed. Etomoxir causes insulin resistance with severe intramyocellular lipid accumulation in mice subjected to 4 weeks of High-Fat Diet (HFD)-feeding [7]. To overcome potential non-specific issues related to pharmacological studies, we recently studied a specific genetic mouse model with heterozygous *Cpt1b*^+/−^ knockout and provided evidence for the first time, supporting the insulin sensitizing effect of CPT1 inhibition [8]. We showed that partial CPT1b deficiency protected against HFD-induced insulin resistance in mice fed a HFD for up to 5 months without significant accumulation of fatty acid-derived metabolites in skeletal muscle. This was also the longest observation period compared to any other studies. Therefore, CPT1b inhibition appeared to be a potentially promising therapeutic approach against diet-induced insulin resistance and onset of diabetes.

Maintaining insulin sensitivity via chronically inhibiting mitochondrial fatty acid oxidation can result in lipid metabolite accumulation in the cytoplasm (reviewed in [9]). Although incompletely understood, intramyocellular accumulation of lipids may be associated with insulin resistant states. It is important to clarify whether a prolonged CPT1b inhibition could eventually lead to insulin resistance due to a potential intramyocellular accumulation of certain lipid intermediates. To test this hypothesis, we extended the HFD feeding in *Cpt1b*^+/−^ mice until the insulin sensitive phenotype failed, and we found that 7 months of HFD feeding did reverse the beneficial effects of CPT1b inhibition.

## Methods

### Animals

Heterozygous *Cpt1b*^+/−^ knockout mice and their Wild-Type (WT) littermates on C57BL/6J background were used [10]. All male mice were kept on a 12-hour/12-hour light/dark cycle in temperature-controlled rooms and had *ad libitum* access to water and standard rodent diet (Harlan Laboratories 7017 NIH-31 Mouse/Rat Sterilizable Diet). Mice (4 weeks old, male) of HFD feeding groups were given *ad libitum* access to HFD (60% kcal% fat) (Research Diets D12492) and water. All experimental procedures were conducted in accordance with the Guide for Care and Use of Laboratory Animals and were approved by the Institutional Animal Care and Use Committee of the University of Alabama at Birmingham (UAB).

### Body Composition Analysis

Fat and lean mass were measured *in vivo* using a quantitative magnetic resonance imaging system (QMR, EchoMRI™ 3-in-1, Echo Medical System, Houston, TX, USA) at UAB Small Animal Physiology Core as previously reported [11].

### Hyperinsulinemic Euglycemic Clamp Study

Procedures of hyperinsulinemic-euglycemic clamp in conscious mice were conducted as previously reported [8]. Five days after catheter implantation on right jugular vein surgery, mice were fasted for 5 hrs in a cage and placed in a rat-size restrainer with its tail taped for the blood glucose measurement using a Contour glucometer (Bayer). A catheter was connected to a CMA 402 syringe pump (CMA Microdialysis, Stockholm, Sweden). [6-^3^H]-glucose was infused at 0.05 µCi/min for 120 minutes without insulin and then infused at 0.1 µCi/min with insulin (Humulin R, Eli Lilly 2.5 mU kg^−1^ min^−1^) for 2 hrs. Blood glucose was maintained at 145 – 155 mg/dL by adjusting the 20 % glucose infusion rate. 13 µCi 2-[^14^C]-deoxy-D-glucose was bolus injected 40 minutes before the end of the 120 minute euglycemic clamp. At the end of the clamp study, mice were euthanized, and tissues were harvested, and snap frozen in liquid nitrogen. The plasma glucose level was measured using an Analox GM7 Micro-Stat Analyzer (Analox Instruments, London, UK). To determine tissue-specific [^14^C]-2DG uptake, supernatants of tissue homogenates were passed through AG 1-X8 resin column (BIO-RAD) followed by washing with water, and the eluted [^14^C]-2DG-6-phosphate was quantified using liquid scintillation counter [12].

### Lipid Measurements

Frozen gastrocnemius muscles were pulverized using a pulverizor (Bio Spec Products Inc.) in liquid nitrogen and weighed. For the Non-Esterified Fatty Acids (NEFA) and Triglyceride (TAG) assay, lipids were extracted using the Bligh & Dyer method [13]. The organic phase was dried at 50°C and reconstituted in 0.5% Triton X-100 solution. NEFA and TAG were measured using a NEFA-HR Kit (Wako) and a Triglyceride Quantification Kit (BioVision K622-100). For the acylcarnitine assay, 6 volumes of 80% acetonitrile were added to pulverized tissue weight (about 50 mg). Tissue mixtures were sonicated 10 times, and centrifuged at 12,000 rpm 10 min at 4°C. The isolated supernatants were then dried under a stream of nitrogen at 40 °C and resuspended in 100 µl of 50% acetonitrile. The acylcarnitine content was measured by using electrospray ionization tandem mass spectrometry [14]. Ceramide content was measured by using high-performance liquid chromatography/mass spectrometry in the Medical University of South Carolina Lipidomics Core as previously described [15]. Analytical results were normalized to total protein.

### Western Blot

Frozen gastrocnemius muscles were homogenized using a pestle pellet homogenizer in a buffer (50 mM Tris HCl pH 6.8, 1% SDS, 2.5 mM DTT, 10% glycerol). The protein concentration of the supernatant was measured by using a Modified Lowry Protein Assay Kit (Pierce #23240). Primary antibodies were purchased from Cell Signaling: pAKT Ser473 (#9271), AKT (#9272), phospho-p44/42 MAPK (#9102), and p44/42 MAPK (#9101). HRP-conjugated secondary antibodies were from Santa Cruz Biotechnology. Western blot images were taken and quantified using ChemiDoc MP System (BIO-RAD, Hercules, CA, USA).

### Statistical analysis

GraphPad Prism *5* software was used to conduct a Two-tailed Student’s *t*-test. Differences between groups were regarded significant at p<0.05 probability level. Data were expressed as the mean ± SE.

## Results

### Exacerbated insulin resistance in *Cpt1b*^+/−^ mice after 7 months of HFD feeding

**Figure 1:**
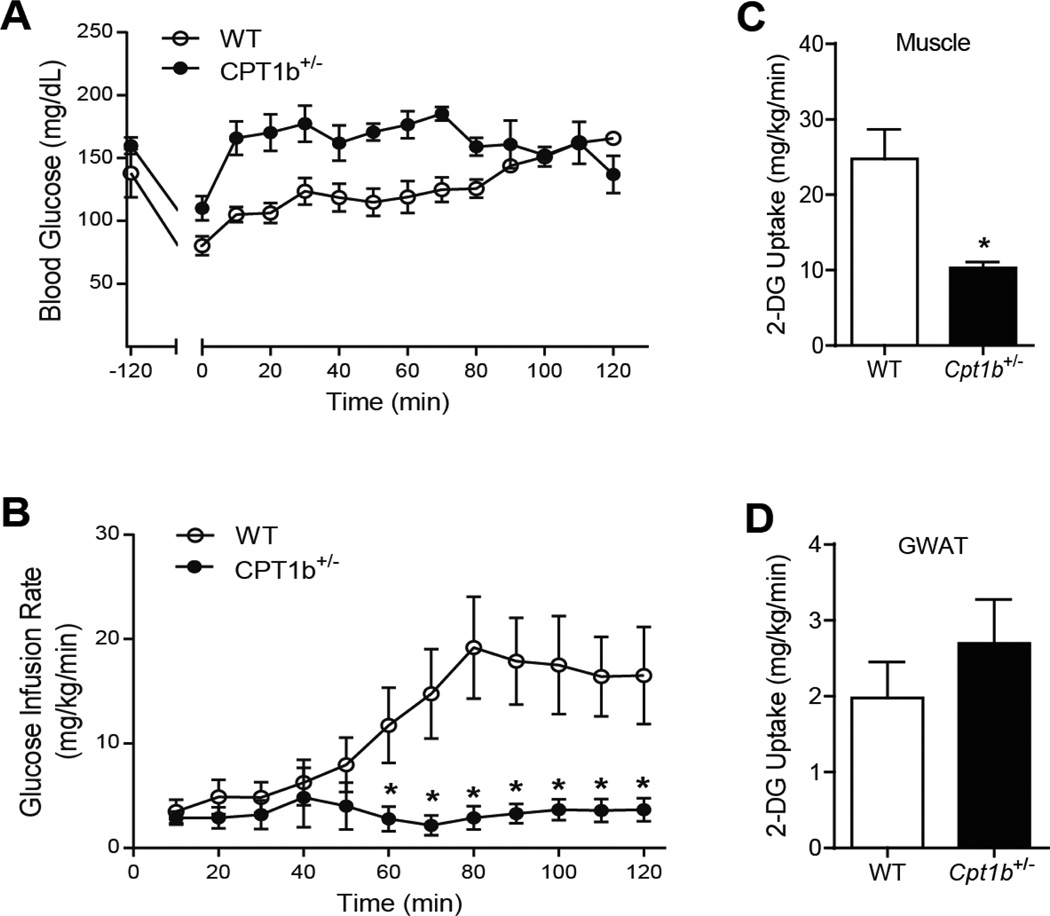
Hyperinsulinemic euglycemic clamp study at 7 months after HFD feeding. (A) Blood glucose level during insulin clamp, (B) Glucose Infusion Rate (GIR), (C) glucose uptake into gastrocnemius muscle, (D) GWAT. *p<0.05, ** p<0.01, n=5 per group.

After 7 months of HFD feeding, we performed hyperinsulinemic-euglycemic clamp studies on *Cpt1b*^+/−^ mice and WT littermates. Strikingly, *Cpt1b*^+/−^ mice showed severe insulin resistance compared to WT mice. The glucose infusion rate (Figure 1B) for maintaining euglycemia was much lower in *Cpt1b*^+/−^ than in WT mice. Minimal amount of glucose infusion immediately raised blood glucose level within 10 minutes after infusion started, clearly indicating whole body insulin resistance in *Cpt1b*^+/−^ mice (Figure 1A). Skeletal muscle-specific glucose uptake (Figure 1C) was substantially decreased in *Cpt1b*^+/−^ mice compared to WT mice (p<0.05), whereas gonadal white adipose tissue (GWAT)-specific glucose uptake was not different between the two groups (Figure 1D). These results demonstrate that prolonged partial *Cpt1b* deficiency reverses the insulin sensitizing effects, especially in skeletal muscle.

### *Cpt1b*^+/−^ mice gain less weight under the prolonged HFD feeding condition

**Figure 2:**
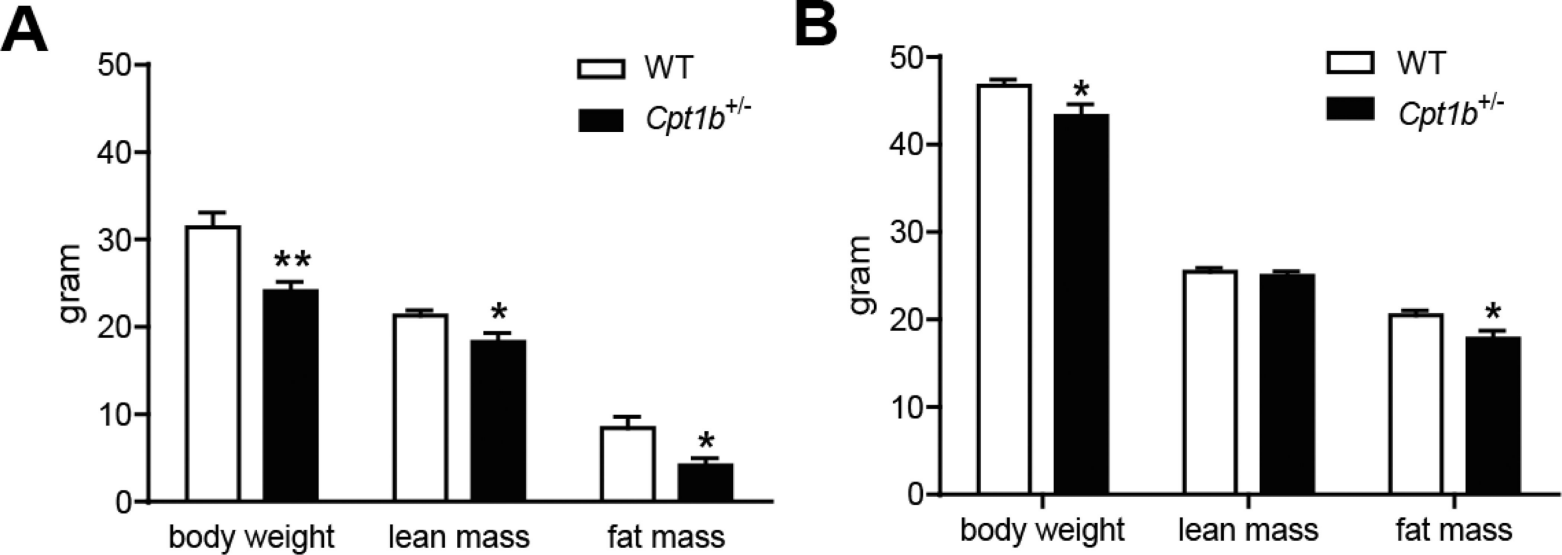
Body composition of mice of HFD feeding. In vivo quantitative magnetic resonance imaging system was used to measure lean and fat mass. (A) 5 months after HFD feeding, (B) 7 months after HFD feeding. *p<0.05, n= 8-7 per group.

Body composition analysis using QMR revealed that *Cpt1b*^+/−^ mice had much lower body weight (30% lower than WT mice, p<0.01), lean mass (10% lower than WT mice, p<0.05), and fat mass (50% lower than WT mice, p<0.05) until 5 month of HFD feeding (Figure 2A). After 7 month of HFD feeding, the body weights of *Cpt1b*^+/−^ mice remained about 10% lower than that of WT mice (Figure 2B). Lean mass became identical between the *Cpt1b*^+/−^ and WT mice, whereas fat mass was still lower in *Cpt1b*^+/−^ mice than in WT mice (p<0.05) (Figure 2B). These data indicate that *Cpt1b*^+/−^ mice remained at a lower body weight due to less fat mass than that of the WT mice in response to prolonged HFD feeding.

### Impaired insulin signaling in the muscle of *Cpt1b*^+/−^ mice after 7 months of HFD feeding

**Figure 3:**
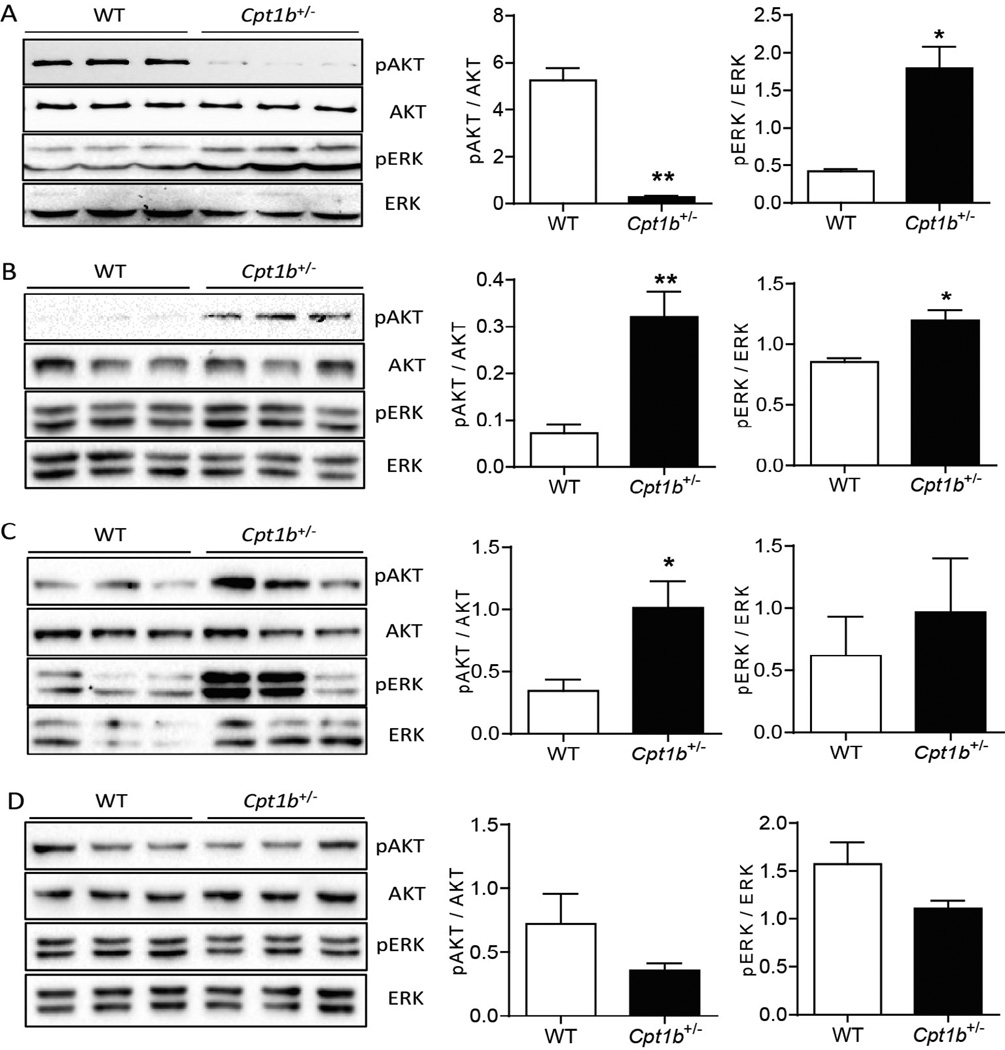
Insulin signaling in different tissues at 7 months after HFD feeding. Tissue samples from insulin clamp were subjected to the Western blot analysis detecting phosphorylation Ser473 AKT, Thr202/Tyr204 p44/42 MAPK (ERK1/2), and total proteins of each target. (A) gastrocnemius muscle, (B) liver, (C) heart, (D) GWAT. *p<0.04, n=4 per group.

We next assessed insulin signaling status in insulin-responsive peripheral tissues from the insulin clamp study using Western blot analysis. As predicted from the hyperinsulinemic-euglycemic clamp study the phosphorylation of AKT at Ser473 was substantially reduced in *Cpt1b*^+/−^ mice muscle (p<0.01), while phosphorylation of p44/42 MAPK (ERK) was similar in muscle of both mouse groups (Figure 3A). Conversely the phosphorylation of AKT at Ser473 was substantially higher in liver of *Cpt1b*^+/−^ mice than in WT mice (Figure 3B) and hearts (Figure 3C), but not in GWAT (Figure 3D). Similarly the phosphorylation of MAPK (ERK) at Thr202/Tyr204 was increased in both liver and muscle samples from *Cpt1b*^+/−^ mice compared with those of WT mice (Figure 3A and B). However, no difference could be detected in the phosphorylation of MAPK (ERK) at Thr202/Tyr204 in cardiac muscle and GWAT from *Cpt1b*^+/−^ mice compared with WT mice (Figure 3C and D). Therefore, these data indicate that the severe insulin resistance of *Cpt1b*^+/−^ mice subjected to a 7 month HFD feeding was specific to skeletal muscle, but not other insulin-responsive tissues such as liver, heart, or GWAT.

### Accumulation of lipid intermediates in the muscle of *Cpt1b*^+/−^mice after 7 months of HFD feeding

**Figure 4:**
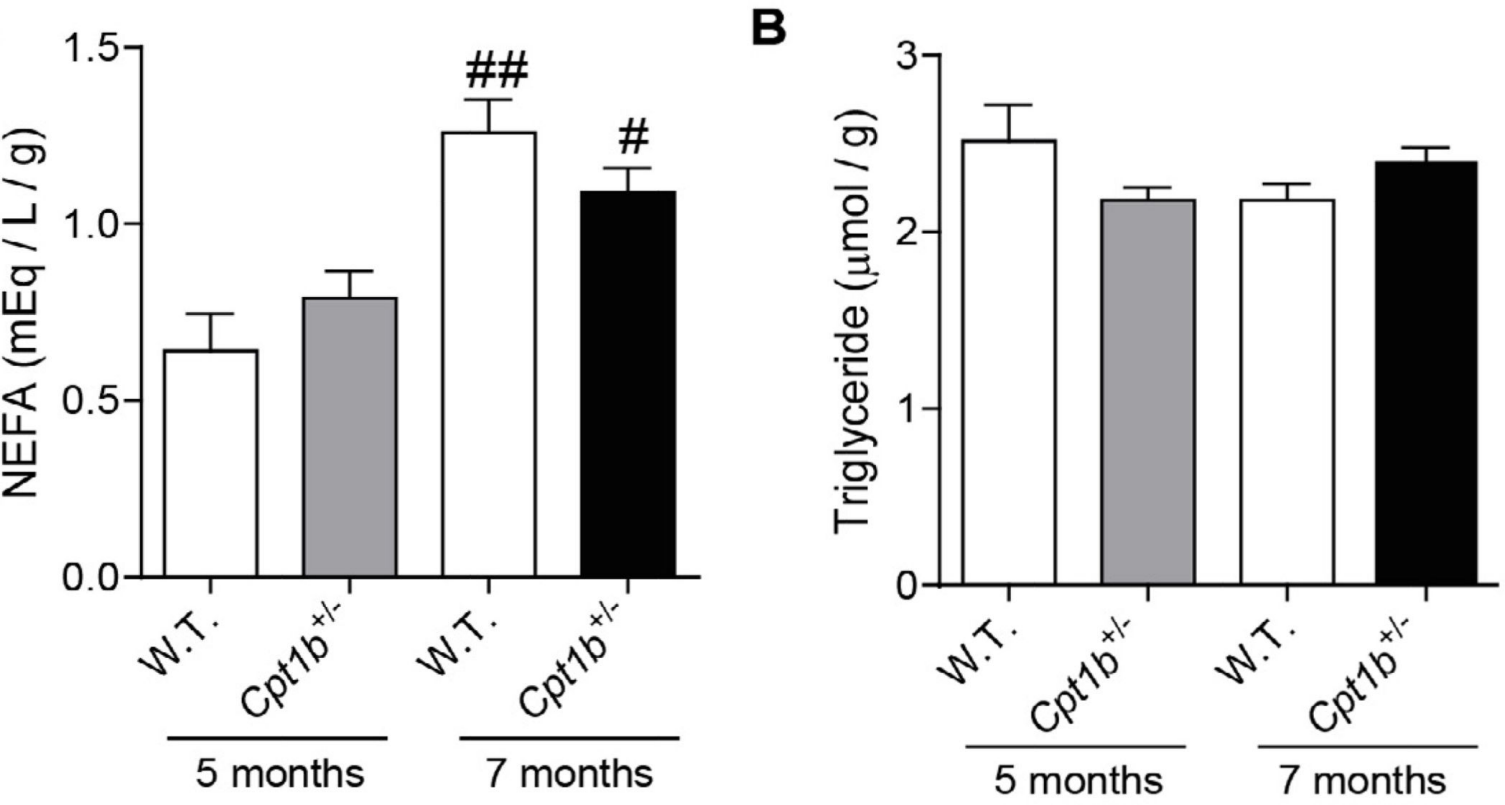
Lipid contents in the gastrocnemius muscle at different time points after HFD feeding. Gastrocnemius muscle samples were used for the lipid extraction. Lipid contents were measured using (A) NEFA-HR Kit (Wako) for NEFA, (B) Triglyceride Quantification Kit (BioVision K622-100) for triglyceride. *p<0.05 WT vs. *Cpt1b*^+/−^, n=5 per group.

**Figure 5:**
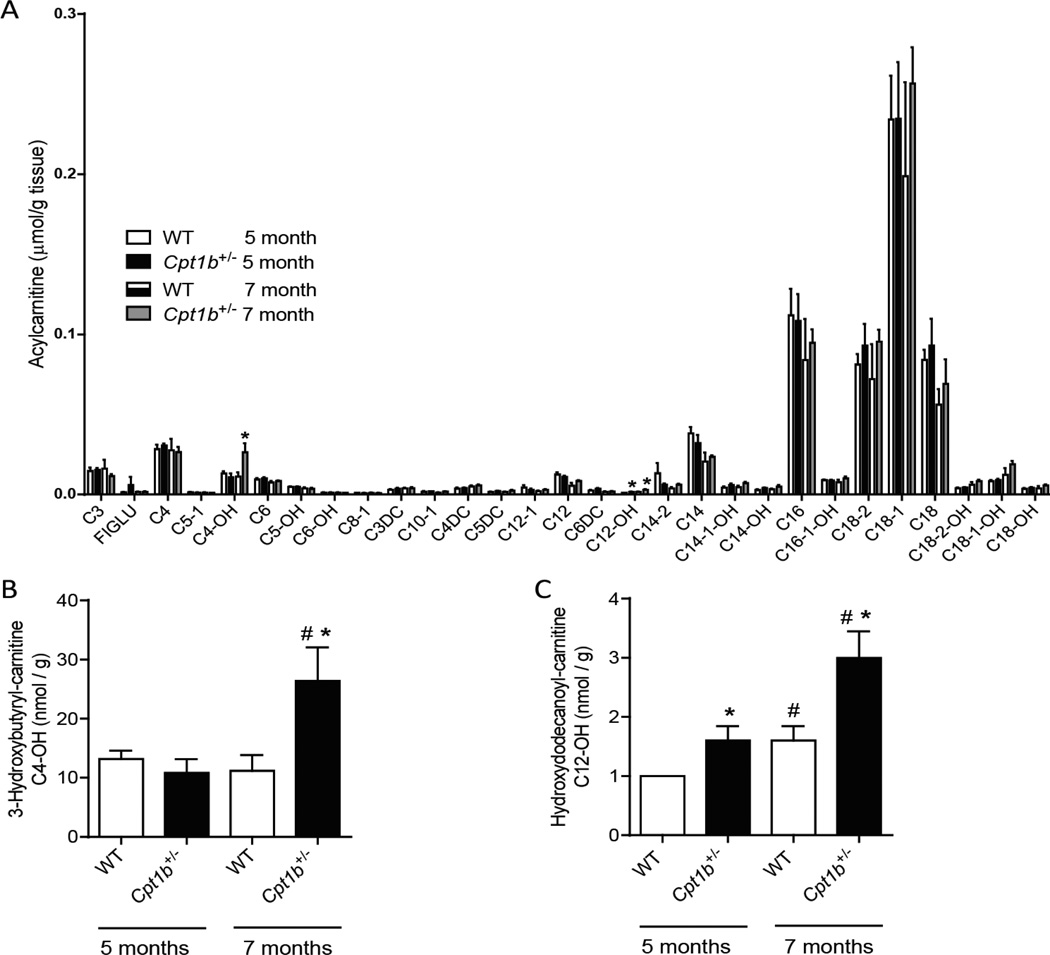
Acylcarnitine contents in the gastrocnemius muscle at different time points after HFD feeding. Acylcarnitine contents in the gastrocnemius muscle samples from 5 months and 7 months after HFD fed mice were measured using electrospray ionization tandem mass spectrometry. (A) acylcarnitine profile, (B) 3-hydroxybutyryl-acylcarnitine (C4-OH), (C) hydroxydodecanoyl-acylcarnitine (C12-OH). #p<0.01, ## p<0.05 between different time points within the same strain mice, *p<0.05 WT vs. *Cpt1b*^+/−^ within the same time point, n=5 per group.

**Figure 6:**
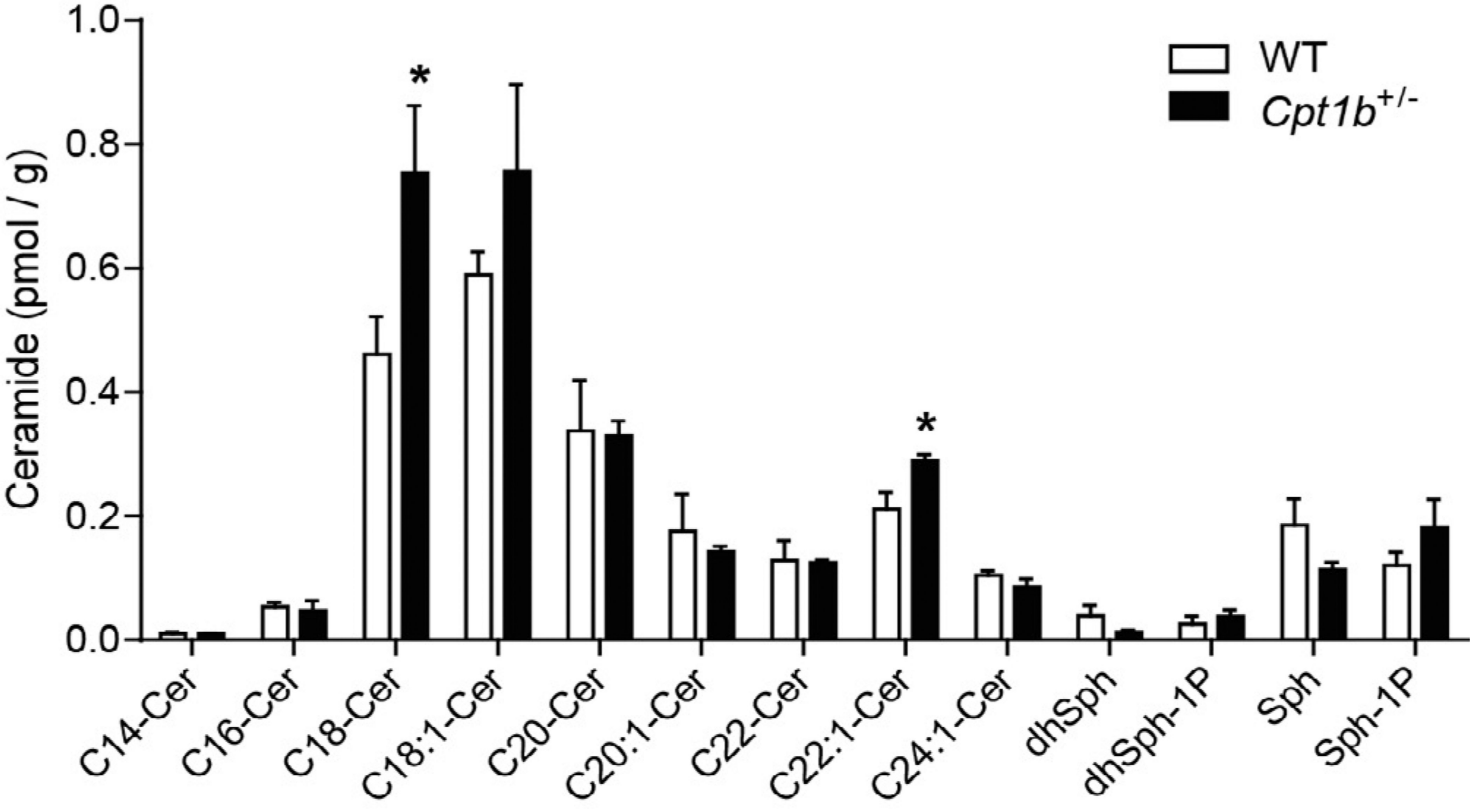
Ceramide profile in the gastrocnemius muscle. Ceramide profile in the gastrocnemius muscle from HFD-fed mice at 7 months using high-performance liquid chromatography/mass spectrometry. *p<0.05 WT vs. *Cpt1b*^+/−^, n=5 per group.

To gain further insights into the differences in skeletal muscle between *Cpt1b*^+/−^ and WT mice, we analyzed the lipid contents of skeletal muscle samples obtained at two different time points. The skeletal muscle samples from 5 months of HFD feeding represent insulin sensitive *Cpt1b*^+/−^ mice and samples from 7 months of HFD feeding represent insulin resistant *Cpt1b*^+/−^ mice. While non-esterified fatty acids were significantly increased after 7 months of HFD feeding in both WT and *Cpt1b*^+/−^ mice compared to those at 5 months of HFD feeding, there were no differences in NEFA contents between the two groups after 7 months (Figure 4A). There was no significant difference in the triacylglycerol contents between the two groups of mice (Figure 4B). The acylcarnitine profiles of the two groups were largely similar (Figure 5A), but 3-hydroxybutyryl-carnitine (C4-OH) content was substantially increased only in *Cpt1b*^+/−^ muscle at 7 months after HFD feeding (p<0.05) (Figure 5B). Hydroxydodecanoyl-carnitine (C12-OH) content was also substantially increased at 7 months of HFD-feeding compared to 5 months of HFD feeding (p<0.05). C12-OH content was also increased in *Cpt1b*^+/−^ compared to WT mice at both time points of 5 months and 7 months (Figure 5C). In addition, we evaluated 13 different ceramide species. Among these, C18- and C22:1-ceramide contents were substantially increased in *Cpt1b*^+/−^ mice compared to WT mice (p<0.05) (Figure 6). Therefore, the above results indicate that accumulated C4-OH acylcarnitine and long-chain ceramide in *Cpt1b*^+/−^ mice muscle after prolonged HFD feeding may account for the impaired insulin signaling.

## Discussion

CPT1, a “rate-controlling” element of FAO, has been studied for decades, and numerous chemical compounds modulating its activity have been developed, although the potential benefits of its activation or inhibition are still arguable (reviewed in [16]). Three CPT1 isoforms are located on the mitochondrial outer membrane. CPT1b is highly expressed in skeletal muscle, heart, and adipose tissues. The liver isoform (CPT1A or CPTI-L) is expressed in multiple tissues except for skeletal muscle and adipose tissues, whereas CPT1c is mainly expressed in brain and testis [4]. Previously, we found that *Cpt1b*^+/−^ mice were protected against HFD-induced insulin resistance in mice with up to 5 months of HFD feeding. However, in the current study we report that *Cpt1b*^+/−^ mice developed severe insulin resistance after 7 months of HFD feeding. Therefore, between the 5 and 7 months period something was significantly altered, leading to impaired insulin sensitivity of *Cpt1b*^+/−^ mice. Interestingly, body weights and fat mass of *Cpt1b*^+/−^ mice were still lower than that of WT littermates, despite the presence of a severe insulin resistant state, suggesting that specific factors (rather than obesity per se) in skeletal muscle may cause the impaired insulin sensitivity in *Cpt1b*^+/−^ mice after prolonged exposure to the HFD feeding. Lipid metabolite assays of skeletal muscle revealed that neither non-esterified free fatty acid nor triglyceride was substantially increased in the *Cpt1b*^+/−^ muscle at the later time point. Studies have suggested that increased triglyceride accumulation in muscle is associated with insulin resistance in rodents and humans (reviewed in [17]), but increased triglyceride concentration could be a surrogate marker of other lipid metabolites accumulating in peripheral tissues. One study found that the intramyocellular triglyceride level is similar between obese, type 2 diabetic, and non-diabetic human subjects, but only the type 2 diabetic subjects show insulin resistance [18]. Because *Cpt1b*^+/−^ mice exhibited similar levels of NEFA and triglyceride in skeletal muscle as compared to WT littermates at both time points, it is unlikely that intramyocellular NEFA and/or triglyceride accumulation plays a role in the reversal of insulin sensitivity.

Since 3-hydroxybutyryl-carnitine (C4-OH) was increased in the *Cpt1b*^+/−^ muscle, we speculate that increased acylcarnitines in the skeletal muscle may be one of the factors causing severe insulin resistance of *Cpt1b*^+/−^ mice. Hydroxybutyryl-carnitine can be produced from either intermediates of fatty acid β-oxidation or ketone body metabolism in human skeletal muscle [19]. Ketone bodies may inhibit insulin stimulated glucose uptake in cultured adult cardiomyocytes by affecting AKT activation [20]. While we did not measure the plasma ketones, the phenotypic change of *Cpt1b*^+/−^ mice is likely not due to diabetic ketoacidosis, because ketoacidosis should have caused systemic effects rather than skeletal muscle-specific insulin resistance [20]. Interestingly, one study found that only 3-hydroxybutyryl-carnitine among 36 acylcarnitine species was decreased in the skeletal muscle of HFD-fed rats, which shows improved insulin sensitivity with hepatic malonyl-CoA decarboxylase (MCD) overexpression [21]. HFD-fed *Mcd*^−/−^ null mice show improved insulin sensitivity with significantly decreased acylcarnitine species in the skeletal muscle by releasing malonyl-CoA-mediated CPT1 suppression and subsequently shifting substrate oxidation from fatty acids to glucose [22]. Both cases support the concept that decreased intramyocellular acylcarnitine can ameliorate insulin resistance, which is consistent with the significantly increased acylcarnitine level in the muscle of insulin resistant *Cpt1b*^+/−^mice after prolonged HFD feeding.

It is possible that other lipid intermediates (e.g., diacylglycerol and acyl-CoA) may also be involved in the development of insulin resistance in *Cpt1b*^+/−^ mice (reviewed in [23]). One study showed that acute lipid infusion into rats caused insulin resistance without intramuscular ceramide accumulation, while acyl-CoA and diacylglycerol were significantly increased in skeletal muscle [24]. This mechanistic approach may have provided important clues, but the acute response within several hours may not exactly reflect insulin resistance in a chronic disease state. On the other hand, studies have shown that ceramide levels are increased in the skeletal muscle of insulin resistant rodents and humans (reviewed in [25]). Significant accumulation of ceramide was observed in C2C12 myotubes treated with saturated fatty acids for 16 hours, which also showed significantly suppressed phosphorylation of AKT without affecting its upstream insulin signaling molecules [26]. Ceramide directly interferes with the translocation of AKT to the plasma membrane in L6 myotubes [27], suppresses GLUT4 gene expression in 3T3L1 adipocytes, and inhibits glucose transport in L6 myotubes [28,29]. In a human study, all ceramide species were significantly increased in the skeletal muscle of insulin resistant subjects [30]. Another human study also found that total ceramide content was negatively correlated with insulin sensitivity under a lipid infusion condition during hyperinsulinemic euglycemic clamp. Palmitic acid (C16:0)-, stearic acid (C18:0)-, and linoleic acid (C18:2)-ceramide concentrations showed strong inverse correlation with insulin sensitivity [31]. We found that C18-ceramide and C22:1-ceramide was significantly increased in skeletal muscle from *Cpt1b*^+/−^ mice. Regarding the aforementioned aspects of ceramides, this could be another important factor causing severe insulin resistance in *Cpt1b*^+/−^ mice after 7 months of HFD feeding. Our Western blot analysis revealed that the phosphorylation of AKT at Ser473 was markedly decreased in the skeletal muscle but not in other tissues, suggesting that the severe insulin resistance may be attributed to impaired phosphorylation of AKT in skeletal muscle. Skeletal muscle comprises 40% of total body mass and 80% of insulin-stimulated glucose disposal [32]. Our current study suggests that glucose catabolism by muscle is a crucial factor determining whole body insulin sensitivity. Furthermore, CPT1b is the CPT1 isoform that plays a crucial role in maintaining lipid homeostasis and therefore insulin sensitivity in skeletal muscle. However, the unanswered question remains as to the mechanism for the accumulation of these lipid intermediates in the skeletal muscle of *Cpt1b*^+/−^ mice only after the prolonged HFD feeding. This appears to be a key consideration for the reversal of insulin sensitivity in skeletal muscle with partial CPT1b deficiency. We speculate that there must be a tipping point whereby these accumulated lipid metabolites exceed a threshold, leading to severe insulin resistance between the time points of 5 months and 7 months in *Cpt1b*^+/−^ mice.

There are a few limitations in our current study. Prolonged FAO inhibition in CPT1b–expressing organs of *Cpt1b*^+/−^ mice resulted in severe insulin resistance only in skeletal muscle, but not other tested organs. The exact mechanism underlying this phenomenon remains unknown, but one of the possible reasons could be that CPT1b in skeletal muscle plays a key role in regulating systemic glucose and fatty acid homeostasis. Our results still provide valuable evidence supporting the significance and major influence of skeletal muscle insulin sensitivity on overall systemic insulin sensitivity. Another possibility is a potential compensation by CPT1a in other organs; CPT1a expression is detected at a low level in heart, white adipose tissue, and brown adipose tissue [16]. Our data clearly prove that the phosphorylation of AKT at Ser473 is significantly suppressed in *Cpt1b*^+/−^ muscle, whether other alterations in insulin signaling of skeletal muscle remain to be determined. Due to the difficulties involved in an animal study using this prolonged HFD feeding protocol, we have not been able to conduct experiments in a larger scale. Further study is necessary to fully explore the detailed mechanism leading to the transition from the beneficial to detrimental state. As summarized in Table 1 based on the previous and the current study, the current study is sufficient to identify that the accumulation of fatty acid related-metabolites, such as acylcarnitines and ceramides, in skeletal muscle plays a key role in this unwanted transition. Therefore, these two studies collectively provide important new insights into the explanations as to why previous studies on this subject have so many conflicting results.

In conclusion, the recent pharmaceutical model with oxfenicine inhibition of CPT-1 in mice and our previous *Cpt1b*^+/−^ genetic mouse model studies support that CPT1b restriction is a good therapeutic approach against diet-induced insulin resistance. Furthermore, our current study reveals an important finding that the optimum treatment time period must be carefully tested to avoid lipid metabolite accumulation to the point of causing insulin resistance in skeletal muscle.

## Figures and Tables

**Table 1:** Summary of phenotypic changes during high fat diet-fed condition, = indicates similar trend, < indicates increased in *Cpt1b*^+/−^ mice, > indicates decreased in *Cpt1b*^+/−^mice, ↓ indicates decreases during designated period in that strain.

Time points		Baseline to HFD 5 months		HFD 5 to 7 months	
**Mouse strains**		**WT**	***Cpt1b***^+/−^	**WT**	***Cpt1b***^+/−^
**Body weight**		>		>	
**Lean mass**		>		=	
**Fat mass**		>		>	
**Glucose tolerance**		↓	=	N/A	N/A
**Insulin tolerance**		↓	=	↓	↓↓
**Insulin signaling** **integrity**	**Skeletal muscle**	<		>	
	**Liver**	<		<	
	**Heart**	<		<	
	**Fat**	>		>	
**Lipid metabolites in** **skeletal muscle**	**NEFA**	=		=	
	**TAG**	=		=	
	**C4-OH carnitine**	=		<	
	**C18, C22:1-ceramide**	**N/A**		<	
